# Association Between the Coronary Sinus Ostial Size and Atrioventricular Nodal Reentrant Tachycardia in Patients With Pulmonary Arterial Hypertension

**DOI:** 10.3389/fphys.2021.790077

**Published:** 2022-01-21

**Authors:** Lei Ding, Sixian Weng, Zhengqin Zhai, Bin Zhou, Yingjie Qi, Fengyuan Yu, Hongda Zhang, Shu Zhang, Min Tang

**Affiliations:** ^1^Department of Cardiology, State Key Laboratory of Cardiovascular Disease, National Center for Cardiovascular Diseases, Fuwai Hospital, Chinese Academy of Medical Sciences and Peking Union Medical College, Beijing, China; ^2^Department of Cardiology, Laboratory of Heart Center, Zhujiang Hospital, Southern Medical University, Guangzhou, China

**Keywords:** pulmonary arterial hypertension, atrioventricular nodal reentrant tachycardia, coronary sinus ostium, radiofrequency ablation, risk factor

## Abstract

**Aims:**

The incidence of atrioventricular nodal reentrant tachycardia (AVNRT) is higher in pulmonary arterial hypertension (PAH) patients than in the general population. AVNRT is reportedly associated with a larger coronary sinus (CS) ostium (CSo). However, the correlation between AVNRT and CSo size in PAH patients is poorly investigated. We aimed to investigate the impact of CSo size on AVNRT and identify its risk factors in PAH.

**Methods and Results:**

Of 102 PAH patients with catheter ablation of supraventricular tachycardia (SVT), twelve with a confirmed AVNRT diagnosis who underwent computed tomographic angiography were retrospectively enrolled as the study group. The control group (PAH without SVT, *n* = 24) was matched for sex and BMI at a 2:1 ratio. All baseline and imaging data were collected. Mean pulmonary artery pressure was not significantly different between the two groups (65.3 ± 16.8 vs. 64.5 ± 17.6 mmHg, *P* = 0.328). PAH patients with AVNRT were older (45.9 ± 14.8 vs. 32.1 ± 7.6 years, *P* = 0.025), had a larger right atrial volume (224.4 ± 129.6 vs. 165.3 ± 71.7 cm^3^, *P* = 0.044), larger CSo in the left anterior oblique (LAO) plane (18.6 ± 3.3 vs. 14.8 ± 4.0 mm, *P* = 0.011), and larger CSo surface area (2.08 ± 1.35 vs. 1.45 ± 0.73 cm^2^, *P* = 0.039) and were more likely to have a windsock-shape CS (75% vs. 16.7%, *P* = 0.001) than those without AVNRT. A linear correlation was shown between CSo diameter in the LAO-plane and the atrial fractionation of the ablation target for AVNRT (*R*^2^ = 0.622, *P* = 0.012).

**Conclusion:**

Anatomical dilation of the CSo is a risk factor for AVNRT development in patients with PAH.

## Introduction

Patients with pulmonary arterial hypertension (PAH) usually experience supraventricular tachycardia (SVT) due to enlargement of the right atrium caused by increased pulmonary pressure (Ruiz-Cano et al., 2011). Atrial fibrillation (AF), atrial flutter (AFL), and atrioventricular nodal reentrant tachycardia (AVNRT) are the three more common SVTs among PAH patients (Fingrova et al., 2021). Many studies have reported that the incidence of AVNRT in PAH patients is approximately 1.3% (Tongers et al., 2007; Cannillo et al., 2015), which is higher than that in the general population (Brugada et al., 2020).

The coronary sinus (CS) is the largest venous system that receives blood from most cardiac veins and drains into the right atrium (RA). The mechanism of AVNRT is related to the reentrant circuit between the slow and fast pathways located at the peri-CS ostium (Katritsis et al., 2016). Several investigations have reported a significant association between the size of the CS ostium (CSo) and AVNRT in the general population (Doig et al., 1995; Okumura et al., 2004) but little is known about this correlation in PAH patients. We hypothesized that increased pulmonary pressure results in dilation of the CS ostium, which provides the arrhythmia substrate for AVNRT. Therefore, we aimed to investigate the impact of the CSo diameter on AVNRT and identify its risk factors in PAH patients.

## Materials and Methods

### Study Population

In this retrospective, single-center study, the data of 102 PAH patients who underwent catheter ablation of SVT between January 2012 and December 2020 in the Department of Cardiology, Fuwai Hospital were analyzed. Among these individuals, the study population was composed of 12 consecutive patients with PAH and AVNRT who had undergone cardiac electrocardiography (ECG) -gated computed tomography angiography (CTA). For the purpose of comparison, a control group (PAH patients without SVT, *n* = 24) was selected from the same center and matched to the AVNRT patients at a ratio of 2:1 based on sex and BMI. CTA was performed as a preoperative examination. The diagnosis of PAH was based on pulmonary pressure obtained from right heart catheterization and defined as a mean pulmonary arterial pressure ≥ 25 mmHg by the current guideline (Galie et al., 2016). The study protocols were approved by the Ethics Committee of Fuwai Hospital, Chinese Academy of Medical Sciences, and were in accordance with the Declaration of Helsinki.

### Electrophysiological Procedures

All catheters were inserted percutaneously by the Seldinger technique and advanced into position under fluoroscopic guidance. CS cannulation was completed *via* an inferior or superior approach (6-F steerable decapolar catheter; Triguy; APT Medical, China) for all AVNRT patients. Additionally, two quadripolar catheters were placed into the right ventricle and His bundle region. We used a multichannel electrophysiology recorder (Bard Electrophysiology) to record the 12-lead surface ECG and intracardiac electrogram. Mapping was performed using three-dimensional electroanatomic mapping systems (CARTO 3, Biosense Webster, Inc., Diamond Bar, CA, United States; EPD Solutions, Philips, Best, Netherlands).

Each patient with PAH and SVT underwent the standard electrophysiology study including extrastimulation and overdrive pacing of the atrium and ventricle to induce tachycardia and identify its mechanism. The diagnostic criterion of AVNRT was based on the standard procedure (Issa et al., 2019). After the diagnosis of AVNRT was confirmed, slow pathway modification was performed guided by fluoroscopy and/or three-dimensional analysis. The slow pathway at the inferior triangle of Koch was targeted for ablation. The endpoints of the procedure were as follows: (1) elimination of the slow pathway conduction which presented as the absence of AH jump, changes in the anterograde refractory periods and Wenckebach point of the atrioventricular node, and/or ≤ one echo beat during programmed stimulation; and (2) non-inducible AVNRT *via* programmed and burst stimulation at baseline and during isoproterenol infusion (Issa et al., 2019).

The atrial fractionation level was defined as the number of deflections at the ablation site (Jadidi et al., 2012). The method of manual calculation of the number of deflections was defined as in a previous study (Saghy et al., 2012). To avoid measurement bias, we used the average fractionated number of five atrial beats at the ablation site.

### Computed Tomography Angiography Measurements and Morphology of the Coronary Sinus Ostium

**FIGURE 1:**
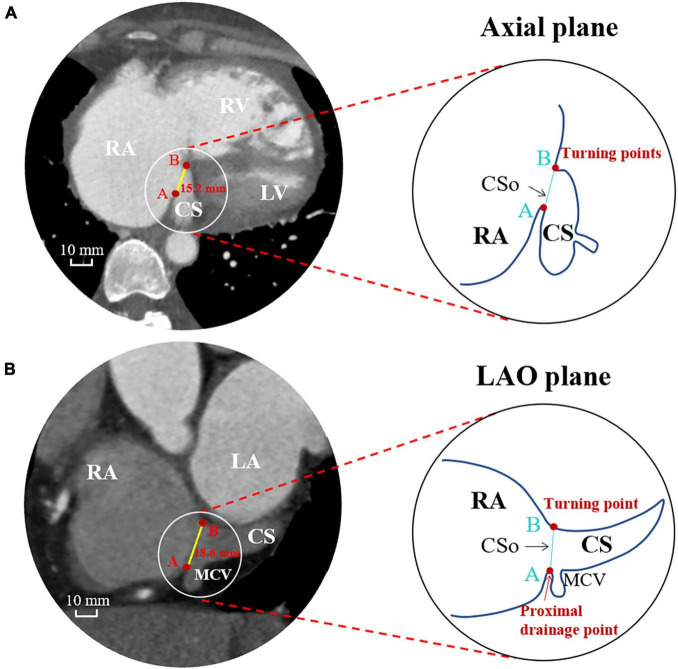
Measurements of the CS ostium in the axial plane **(A)** and LAO plane **(B)** by computed tomographic angiography. CS, coronary sinus; CSo, coronary sinus ostium; LA, left atrium; LAO, left anterior oblique; LV, left ventricle; MCV, middle cardiac vein; RA, right atrium; RV, right ventricle.

All patients underwent cardiac ECG-gated CTA using a 64-slice CT scanner. Every patient was injected with contrast media in an antecubital vein. According to body weight, we injected iohexol 350 mgI/ml (Omnipaque 350, GE Healthcare, United States) at a flow rate of 4.0–5.0 ml/s. For contrast medium enhancement, bolus-tracking was used in a region-of-interest (ROI) at the ascending aorta. When the attenuation threshold of the ROI was more than 100 HU, an autodelay of 6 s was triggered. Axial images were obtained through the lower heart region with a slice thickness of 0.625 mm. Measurements of the CSo diameter and the surface area were performed using a RadiAnt DICOM Viewer workstation (Medixant Inc., Poland) in 75% phase of the cardiac cycle. Atrial volume was obtained by drawing free-hand ROIs on each slice of the atria in the axial plane and automatically calculated by post-processing software (Siemens, Erlangen, Germany). All CT data were measured by two experienced CTA observers. To keep the results comparable and reliable, all measurements were performed in two perpendicular planes, the axial plane and the left anterior oblique (LAO) plane, on multiplanar reconstruction (MPR). The diameter measured in the LAO plane represents the vertical diameter of the CSo. The ostium of the CS is defined as follows: (i) the line connecting the turning points of its nearest proximity to the RA; and (ii) the border of the CSo, which includes the proximal drainage point of the middle cardiac vein (MCV). The maximal diameters of the CSo in the axial plane and LAO plane were documented. Representative measurements of the CSo in two perpendicular planes (axial and LAO) is presented in Figure 1. The surface area of the CSo was measured by stepwise procedures *via* CTA (Supplementary Figure 1). CS morphology is defined (Okumura et al., 2004; Ong et al., 2006) as either windsock shape with tapering of the vessel occurring 10–15 mm distal to the CS ostium or a tubular shape with gradual tapering of the vessel. The morphologic index is defined as the ratio of the diameters in the axial plane and LAO plane.

### Statistical Analysis

SPSS IBM 22 (IBM Co., Armonk, New York, NY, United States) and GraphPad Prism 8.0 (GraphPad Software Inc., La Jolla, CA, United States) were used for statistical analysis and graphing. Continuous variables are presented as the mean ± SD and analyzed by independent-samples *T*-test if normally distributed; otherwise, the Mann–Whitney *U* test was used. Categorical variables are reported as frequencies and percentages and were tested with the χ^2^-test. Variables with a *P* value < 0.05 by univariate analysis and clinically meaningful variables were entered into multivariate logistic regression to assess the independent risk factors for AVNRT in PAH. The interobserver and intraobserver agreement of CTA measurements was evaluated by a correlation coefficient. *P* values < 0.05 were considered indicative of statistical significance.

## Results

### Baseline Characteristics

**FIGURE 2:**
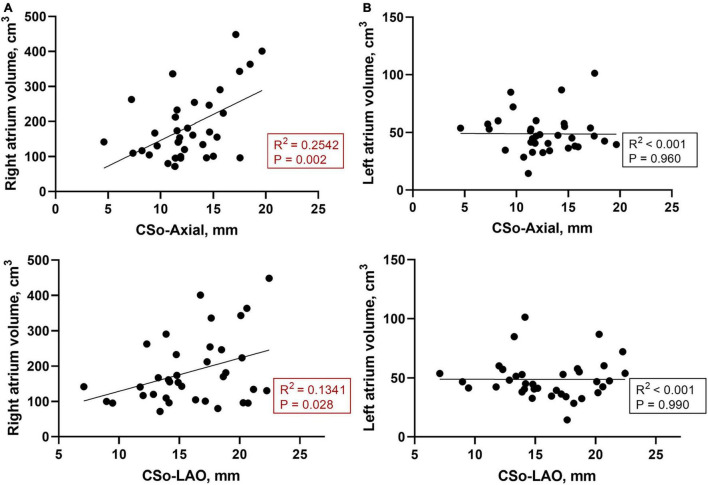
**(A)** Correlation between the RA volume and CS diameter of the LAO and axial plane (*n* = 36); **(B)** Correlation between the LA volume and CS diameter of the LAO and axial planes (*n* = 36). CSo, coronary sinus ostium; LA, left atrium; LAO, left anterior oblique; RA, right atrium.

The baseline characteristics of the 36 patients are listed in Table 1. Overall, we studied 12 PAH patients with AVNRT and 24 PAH patients without AVNRT in a 1:2 ratio in this case-control study. All of the patients in the study group were confirmed to have slow-fast AVNRT by the electrophysiology study and underwent slow pathway modification. All ablation targets were rightward inferior extensions. The mean ages of the subjects were 45.9 ± 14.8 and 32.1 ± 7.6 years in the study and control groups, respectively. There was no difference in sex, BMI, body surface area (BSA) or hypertension history between the two groups. One patient in the case group had a history of atrial septal defect (ASD) and one patient in the control group had a history of patent foramen ovale (PFO), while no other structural heart diseases were recorded in our population. PAH patients with AVNRT tended to have a significantly larger RA volume than those without (224.4 ± 129.6 vs. 165.3 ± 71.7 cm^3^, *P* = 0.044). In addition, we found that patients with a larger CS ostium tended to have a higher RA volume (Figure 2A), while there was no obvious correlation between the size of the CSo and LA volume (Figure 2B). More than 90% of patients in both groups had idiopathic PAH (91.7% vs. 95.8%, *P* = 0.607). Moreover, there was no significant difference in mean PA pressure between the two groups (65.3 ± 16.8 vs. 64.5 ± 17.6 mmHg, *P* = 0.328). As shown in Supplementary Figure 2, we did not find a linear correlation between mean PA pressure and CSo diameter in the case group, control group, or the total patient group. Other details are shown in Table 1.

**TABLE 1:** Baseline characteristics and measurements of the CSo.

Variables	PAH + AVNRT (*n* = 12)	PAH (*n* = 24)	*P* value
Age (years)	45.9 ± 14.8	32.1 ± 7.6	0.025*
Male, *n* (%)	2 (16.7)	4 (16.7)	> 0.999
BMI (kg/m^2^)	21.9 (19.1, 24.4)	23.7 (19.9, 26.5)	0.455
BSA (m^2^)	1.5 ± 0.2	1.6 ± 0.2	0.322
Hypertension, *n* (%)	3 (25.0)	2 (8.3)	0.300
PFO, *n* (%)	0	1 (4.2)	0.473
ASD, *n* (%)	1 (8.3)	0	0.151
LVEDD, mm	39.1 ± 6.5	34.4 ± 5.3	0.042*
LVEF,%	63.6 (59.5, 73.4)	65.0 (63.0, 70.0)	0.675
LAAD, mm	32.0 ± 5.0	29.1 ± 3.4	0.071
IVS, mm	9.1 ± 1.1	8.4 ± 1.3	0.158
RV, mm	34.0 (22.8, 44.3)	32.5 (28.3, 37.5)	> 0.999
RA anteroposterior dimension (mm)	64.4 ± 14.7	58.5 ± 7.6	0.142
RA volume (cm^3^)	224.4 ± 129.6	165.3 ± 71.7	0.044*
LA volume (cm^3^)	57.7 ± 24.7	44.4 ± 8.9	0.095
Etiology of PAH, *n* (%)			
Idiopathic	11 (91.7)	23 (95.8)	0.607
Associated with CHD	1 (8.3)	0	0.151
Pulmonary veno-occlusive disease	0	1 (4.2)	0.473
Mean PAP (mmHg)	65.3 ± 16.8	64.5 ± 17.6	0.328
Mean PAP ≥ 50 mmHg, *n* (%)	10 (83.3)	16 (66.7)	0.293
CSo (Axial plane), mm	14.6 ± 3.6	11.6 ± 3.2	0.027*
CSo (LAO plane), mm	18.6 ± 3.3	14.8 ± 4.0	0.011*
Morphologic index	1.3 ± 0.4	1.3 ± 0.4	0.868

*Data represent the mean ± SD for continuous variables and n (%) for dichotomous variables.*

ASD, atrial septal defect; AVNRT, atrioventricular nodal reentrant tachycardia; BMI, body mass index; BSA, body surface area; CHD, congenital heart disease; CSo, coronary sinus ostium; IVS, interventricular septum; LA, left atrium; LAAD, left atrial anteroposterior dimension; LAO, left anterior oblique; LVEDD, left ventricular end-diastolic diameter; LVEF, left ventricular ejection fraction; PAH, pulmonary arterial hypertension; PAP, pulmonary artery pressure; PFO, patent foramen ovale; RA, right atrium; RV, right ventricular anteroposterior dimension. *P ≤ 0.05.

### Measurements and Morphology of Coronary Sinus Ostium

**FIGURE 3:**
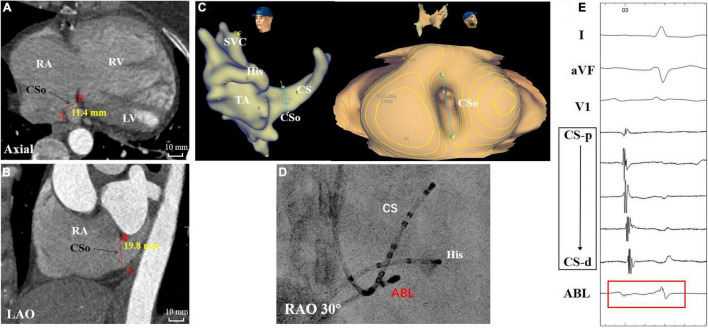
A patient with PAH and AVNRT showed marked vertical dilation of the CSo. **(A,B)** Measurements in the axial and LAO planes by CTA. **(C)** Three-dimensional image and panoramic view of the CSo. **(D)** Fluoroscopy of the ablation target. **(E)** Intracardiac electrogram of the ablation target (red box) during sinus rhythm. ABL, ablation catheter; AVNRT, atrioventricular nodal reentrant tachycardia; CTA, computed tomographic angiography; CS, coronary sinus; CS-d to CS-p: from distal-to-proximal pair of coronary sinus electrode; CSo, coronary sinus ostium; His, His bundle catheter; LAO, left anterior oblique; LV, left ventricle; PAH, pulmonary arterial hypertension; RA, right atrium; RAO, right anterior oblique; RV, right ventricle; SVC, superior vena cava; TA, tricuspid annulus.

**FIGURE 4:**
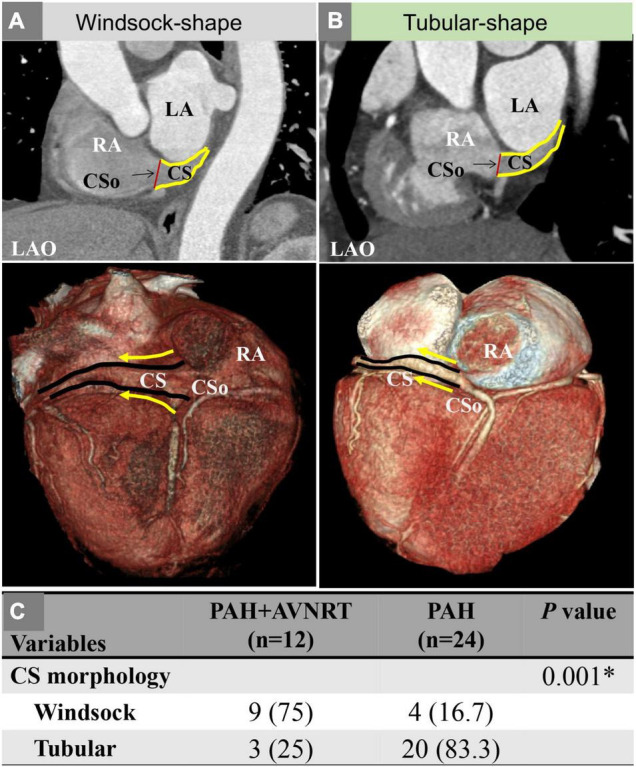
**(A)** A patient with PAH and AVNRT showing a windsock-shaped CS; **(B)** A patient with PAH showing a tubular shape; **(C)** Comparison of the CS morphology between the two groups. AVNRT, atrioventricular nodal reentrant tachycardia; CS, coronary sinus; CSo, coronary sinus ostium; LAO, left anterior oblique; PAH, pulmonary arterial hypertension; RA, right atrium. The solid yellow line represents the outlines of the CS; the solid yellow arrow indicates the direction of the CS.

Table 1 also shows the measurement of the diameter of the CSo in the axial plane and LAO plane. Two observers performed all measurements independently. These measurements were checked for interobserver (Supplementary Figure 3) and intraobserver agreement (Supplementary Figures 4, 5). The interclass correlation coefficient was 0.98 (95% CI 0.96–0.99), the intraobserver correlation coefficient of observer 1 was 0.996 (95% CI 0.993–0.998), and the intraobserver correlation coefficient of observer 2 was 0.994 (95% CI 0.990–0.996), suggesting good agreement. The average CSo diameters measured in the axial plane were 14.6 ± 3.6 and 11.6 ± 3.2 mm in the PAH with AVNRT and PAH without AVNRT groups, respectively. The average diameter in the LAO plane in the study group was larger than that in the control group (18.6 ± 3.3 mm vs. 14.8 ± 4.0 mm, Figure 3). The diameters in both planes showed a significant difference (*P* = 0.027 and *P* = 0.011). Although there was no significant difference in the morphological index between the two groups (1.3 ± 0.4 vs. 1.3 ± 0.4, *P* = 0.868), we found that PAH patients with AVNRT had a significantly larger CSo surface area than those without AVNRT (2.08 ± 1.35 vs. 1.45 ± 0.73 cm^2^, *P* = 0.039). In addition, in the PAH with AVNRT group, the CS of 75% (9/12) of the patients had a windsock appearance, while only 4 patients (16.7%) showed a windsock-shaped CS in the PAH group (*P* = 0.001, Figure 4).

### Electrophysiological Properties and Ablation Outcomes

**FIGURE 5:**
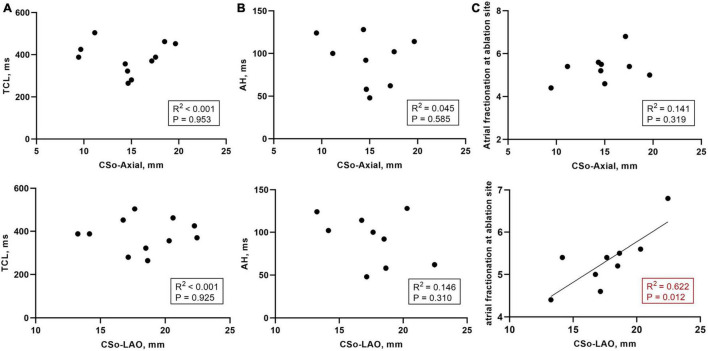
**(A)** Correlation between the TCL (*n* = 11) and CS diameter in the LAO and axial planes; **(B)** Correlation between the AH interval during sinus rhythm (*n* = 9) and CS diameter in the LAO and axial planes; **(C)** Correlation between the atrial fractionation at the ablation site (*n* = 9) and CS diameter in the LAO and axial planes. AH interval, atrial-His interval; CSo, coronary sinus ostium; LAO, left anterior oblique; TCL, tachycardia cycle length.

In PAH patients with AVNRT, the acute success rate was 100%. Ten patients exhibited successful elimination the of slow pathway conduction, and two patients still had a single echo beat. Furthermore, AVNRT could not be induced in any of the patients *via* programmed or burst stimulation at baseline or during isoproterenol infusion. Eleven patients had the data on the AH interval during sinus rhythm, and 9 patients had the data on the tachycardia cycle length (TCL) and the atrial fractionation at the ablation site. We found that the mean values of the AH interval, TCL, and atrial fractionation at the ablation site were 92.0 ± 29.5 ms, 382.8 ± 75.4 ms, and 5.2 ± 1.3 deflections, respectively. In addition, we also investigated the correlation between those values and the CSo dimensions. We found that there was no significant linear correlation between the AH interval or TCL and CS dimensions (*R*^2^ < 0.15, Figures 5A,B). A relatively good correlation was shown between CSo diameter in the LAO- plane and atrial fractionation (*R*^2^ = 0.622, *P* = 0.012, Figure 5C).

### Multivariate Logistic Analysis

**FIGURE 6:**
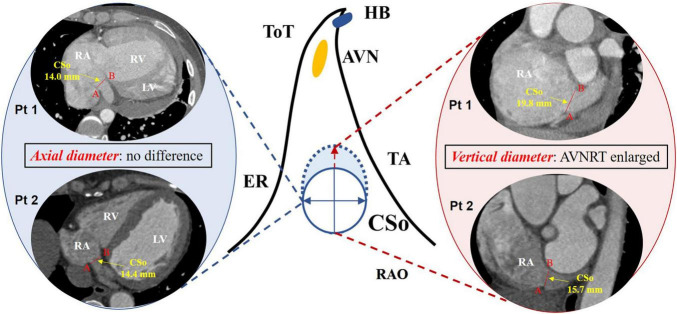
Difference in the CSo diameter in PAH patients with and without AVNRT according to multivariate logistic regression. Left panel: axial diameter of the CSo in PAH patients with AVNRT (Pt1) and without AVNRT (Pt2); middle panel: schemes of the triangle of Koch with a normal (solid line) and an enlarged CSo (dashed line); right panel: vertical diameter of the CSo in PAH patients with AVNRT (Pt1) was larger than that in patients without AVNRT (Pt2). AVN, atrioventricular node; AVNRT, atrioventricular nodal reentrant tachycardia; ER, Eustachian ridge; HB, His bundle; TA, tricuspid annulus; ToT, tendon of Todaro; CSo, coronary sinus ostium; LV, left ventricle; PAH, pulmonary arterial hypertension; Pt, patient; RA, right atrium; RAO, right anterior oblique; RV, right ventricle.

To further explore the correlation between the diameter of the CSo and AVNRT as well as other risk factors for AVNRT development in PAH patients, multivariable logistic regression analysis using a forward stepwise approach was performed. The variables included left ventricular end-diastolic diameter (LVEDD), RA volume, diameter of the CSo in the axial plane and in the LAO plane, and age (Table 2). We found that increased age and diameter of the CSo-LAO-plane (Figure 6) were independent risk factors for AVNRT development in PAH patients.

**TABLE 2:** Univariate and Multivariate analyses of factors associated with AVNRT in PAH patients.

	Univariate analysis		Multivariate analysis	
	OR (95% CI)	*P*	OR (95% CI)	*P*
LVEDD	1.19 (1.02, 1.37)	0.025	1.16 (0.89, 1.51)	0.265
RA volume	1.00 (0.99, 1.01)	0.048	1.01 (0.99, 1.02)	0.149
CSo axial plane, mm	1.38 (1.04, 1.82)	0.026	0.68 (0.37, 1.28)	0.233
CSo LAO plane, mm	1.41 (1.07, 1.85)	0.014	1.44 (1.01, 2.05)	0.042
Age (years)	1.13 (1.03, 1.23)	0.009	1.11 (1.00, 1.23)	0.048

*AVNRT, atrioventricular nodal reentrant tachycardia; CI, confidence interval; CSo, coronary sinus ostium; LAO, left anterior oblique; LVEDD, left ventricular end-diastolic diameter; OR, odds ratio; PAH, pulmonary arterial hypertension.*

*β is the regression coefficient.*

## Discussion

### Major Findings

The main findings of the present study are as follows: (i) PAH patients with AVNRT tend to have a larger RA volume and CSo surface area and are more likely to have a windsock-shaped CS than those without AVNRT; (ii) increased age and diameter of the CSo in the LAO plane were independent risk factors for AVNRT development in PAH patients.

### The Relationship Between the Coronary Sinus Ostium and Atrioventricular Nodal Reentrant Tachycardia in Pulmonary Arterial Hypertension Patients

Atrioventricular nodal reentrant tachycardia is one of the most common SVTs among PAH patients, with an incidence of 35/100,000 person-years. Additionally, some studies have investigated the epidemiological characteristics of AVNRT in PAH patients. According to a cross-sectional study conducted by Tongers et al. (2007), the incidence of AVNRT among 231 PAH patients was approximately 1.3%, ranking third among all types of SVT. Fingrova et al. (2021) found that AVNRT occurred in approximately 1.2% of PAH patients (Cannillo et al., 2015), and they also reported the prevalence of SVT in different subgroups of pulmonary hypertension patients according to etiology. According to the guidelines for pulmonary hypertension (Galie et al., 2016), the clinical classification is intended to categorize multiple clinical conditions into five groups. The prevalence rates of AVNRT in group 1 (including idiopathic, heritable, and drug and toxin induced pulmonary hypertension as well as associated specific diseases), group 4 (chronic thromboembolic pulmonary hypertension), group 5 (pulmonary hypertension with unclear and/or multifactorial mechanisms), and all PAH patients were 0.6, 0.8, 2.7, and 0.6%, respectively. In summary, the incidence or prevalence of AVNRT is much higher in PAH patients than in the general population (Brugada et al., 2020), but none of these studies revealed the cause. In the past few years, studies have investigated the mechanisms of AVNRT in the general population. The circuit of AVNRT proposed by Jackman et al. was located at the peri-CSo, and many other studies have focused on the correlation between the CSo and AVNRT. Doig et al. (1995) measured the CSo diameter by venography in the AVNRT group and control group and proposed that AVNRT patients had significantly larger CSos with a mean maximum diameter of 10.2 mm. Ong et al. (2006) also found that typical AVNRT patients had a larger CSo than atypical AVNRT patients. Another previous study (Ezhumalai et al., 2014)investigated the relationship between AVNRT and the CSo by transesophageal echocardiography (TEE) and concluded that AVNRT patients tend to have a significantly larger CSo diameter (14.1 ± 5 mm). All of the above studies showed that AVNRT patients have a larger CSo diameter. Among patients with PAH, AVNRT usually occurs several years after the onset of increasing pulmonary pressure. Therefore, we hypothesize that elevated pulmonary pressure may change the peri-CSo tissue and trigger AVNRT.

Waligora et al. (2018) enrolled 97 PAH patients and assessed the predictors of newly occurring significant supraventricular arrhythmia, mainly atrial fibrillation and atrial flutter. They found that RA enlargement was associated with an increased prevalence of these SVTs. Similarly, a larger RA volume was observed in the PAH with AVNRT group with a larger CSo in the present study. Thus, we included both the RA volume and the CSo diameters in the multivariate logistic analysis and found that the CSo diameter in the LAO- plane was an independent risk factor. Our study ultimately found that in the PAH population, the CSo was significantly larger in patients with AVNRT than in those without AVNRT, which was in accordance with previous studies (Doig et al., 1995; Ong et al., 2006; Ezhumalai et al., 2014). We also found that the CSo was enlarged to a greater extent along the vertical diameter than along the axial diameter. Elliptical enlargement of the CSo was characteristic of PAH patients with AVNRT. Because of the larger CSo in PAH patients, many studies have proposed various hypotheses. Yuce et al. (2010) indicated that CS dilation may be a part of cardiac remodeling caused by PAH. Another previous study (Isaacs et al., 2009) involved visualization and measurement of the CS on chest CT and showed that CS dilation was associated with increased pulmonary artery pressure. Cetin et al. (2015) also demonstrated through a case-control study that the CS diameter was significantly increased in patients with moderate-to-severe PAH. Therefore, increased pulmonary artery pressure, which enlarges the CSo, may provide a foundation for the development of AVNRT. The circuit of typical AVNRT is formed by the upper fast pathway and lower slow pathway near the CSo, which was proposed by Jackman et al. (2007). We suspected that elliptical enlargement of the CSo provides an anatomic substrate for AVNRT. An enlargement of the ostium stretches the peri-ostial tissue vertically and extends the distance between the slow pathway and fast pathway, eventually resulting in anisotropic conduction (Figure 6). Moreover, a larger CSo could enlarge the circuit of AVNRT and stabilize the reentry.

It is widely recognized that the onset of AVNRT in the general population has a bimodal age distribution as people younger than 20 years and older than 50 years are prone to AVNRT (Brugada et al., 2020). Previous studies (Pentinga et al., 1993; Wu et al., 1993) found that functions of the sinus node and atrioventricular conduction system are decreased in elderly patients and that the effective refractory periods of the right atrium and ventricle are also greater. This may be concerning to the findings in the present study. Generally, the incidence of AVNRT in the early stage of PAH patients should be identical to that in the general population (first incidence peak). As age increases, anatomical and electrical remodeling of the CSo in PAH patients occurs earlier than that in the general population. Therefore, the second peak of AVNRT onset should theoretically be advanced. In actuality, the mean age of the case group (45.9 years) in the present study was younger than the peak age at the second incidence (after 50 years) of AVNRT reported by previous studies. The above hypothesis was proved in our study. For the PAH patients with AVNRT, the mean age was 45.9 ± 14.8 years, which was older than that of those without AVNRT. After adjusting for other potential risk factors using multivariate logistic regression, we concluded that age is another risk factor for AVNRT in PAH patients. We supposed that the CSo diameter increases with age and that the pulmonary artery pressure increases, and thus, the dilated CSo eventually triggers AVNRT through the mentioned mechanism.

## Limitations

First, the small sample size and single-center study design limit the extrapolation of the conclusion. However, considering that PAH is not a common disease and that AVNRT is even rarer, the statistical power was high enough to detect differences in CSo diameter between the two groups. Second, this study was a retrospective case-control study, whereas a prospective cohort study with a larger population is necessary to evaluate the correlation between the CSo diameter and AVNRT in PAH patients. Third, the cardiac cycle may influence the size of the CSo, and we measured the 75% phase in every patient, which guaranteed the consistency of the measurements.

## Conclusion

Anatomical dilation of the CSo is a risk factor for developing AVNRT in patients with PAH as age increases. This morphologic variation may provide an arrhythmia substrate for AVNRT in PAH patients.

## Data Availability Statement

The datasets presented in this article are not readily available because research data is confidential. Data sharing requests are required to meet the policies of the hospital and the funder. Requests to access the datasets should be directed to MT, doctortangmin@yeah.net.

## Ethics Statement

The studies involving human participants were reviewed and approved by Ethics Committee of Fuwai Hospital, Chinese Academy of Medical Sciences. The patients/participants provided their written informed consent to participate in this study.

## Author Contributions

MT and LD conceived and designed the research. MT, H-DZ, F-YY, Y-JQ, Z-ZQ, and BZ conducted the ablation. LD and S-XW analyzed the data and wrote the manuscript. SZ revised the manuscript. All authors read and approved the final manuscript.

## Conflict of Interest

The authors declare that the research was conducted in the absence of any commercial or financial relationships that could be construed as a potential conflict of interest.

## Publisher’s Note

All claims expressed in this article are solely those of the authors and do not necessarily represent those of their affiliated organizations, or those of the publisher, the editors and the reviewers. Any product that may be evaluated in this article, or claim that may be made by its manufacturer, is not guaranteed or endorsed by the publisher.

## Supplementary Material

Supplementary Figure 1 Stepwise procedure of measurement of the CS ostium surface area. **(A)** Adjustment of the blue line parallel to the CS ostium in the axial plane; **(B)** Adjustment of the blue line parallel to the CS ostium in the LAO plane; **(C)** After multiplanar reconstruction **(A,B)**, the CS ostium was at the intersection of the pink line and yellow line in the LPO plane; **(D)** The CS ostium was encircled by the green line, and the surface area in the green circle was automatically calculated by the software (1.185 cm^2^). AVNRT, atrioventricular nodal reentrant tachycardia; CSo, coronary sinus ostium; LA, left atrium; LAO, left anterior oblique; LPO, left posterior oblique; LV, left ventricle; PAH, pulmonary arterial hypertension; AO, aorta; RA, right atrium. The blue, pink, and yellow lines were perpendicular to each other. A solid blue arrow in panel **(A)** represents the projection direction of panel **(B)**; a solid red arrow in panel **(B)** represents the projection direction of panels **(C,D)**.

Supplementary Figure 2 **(A)** Correlation between the mean PA pressure and CS diameter in the LAO and axial planes in all patients (*n* = 36); **(B)** Correlation between the mean PA pressure and CS diameter in the LAO and axial plane in PAH patients with AVNRT (*n* = 12); **(C)** Correlation between the mean PA pressure and CS diameter in the LAO and axial planes (*n* = 24) in PAH patients. LAO, left anterior oblique; PA, pulmonary artery.

Supplementary Figure 3 Bland–Altman plots with 95% confidence intervals comparing measurements between observer 1 and observer 2.

Supplementary Figure 4 Bland–Altman plots with 95% confidence intervals comparing measurements of observer 1 between the first time and the second time.

Supplementary Figure 5 Bland–Altman plots with 95% confidence intervals comparing measurements of observer 2 between the first time and the second time.
