# Supplementary figures and images for: Association Between the Coronary Sinus Ostial Size and Atrioventricular Nodal Reentrant Tachycardia in Patients With Pulmonary Arterial Hypertension
Source: Front Physiol. 2022 Jan 21;12:790077. doi: 10.3389/fphys.2021.790077 (PMC8814530; doi:10.3389/fphys.2021.790077)

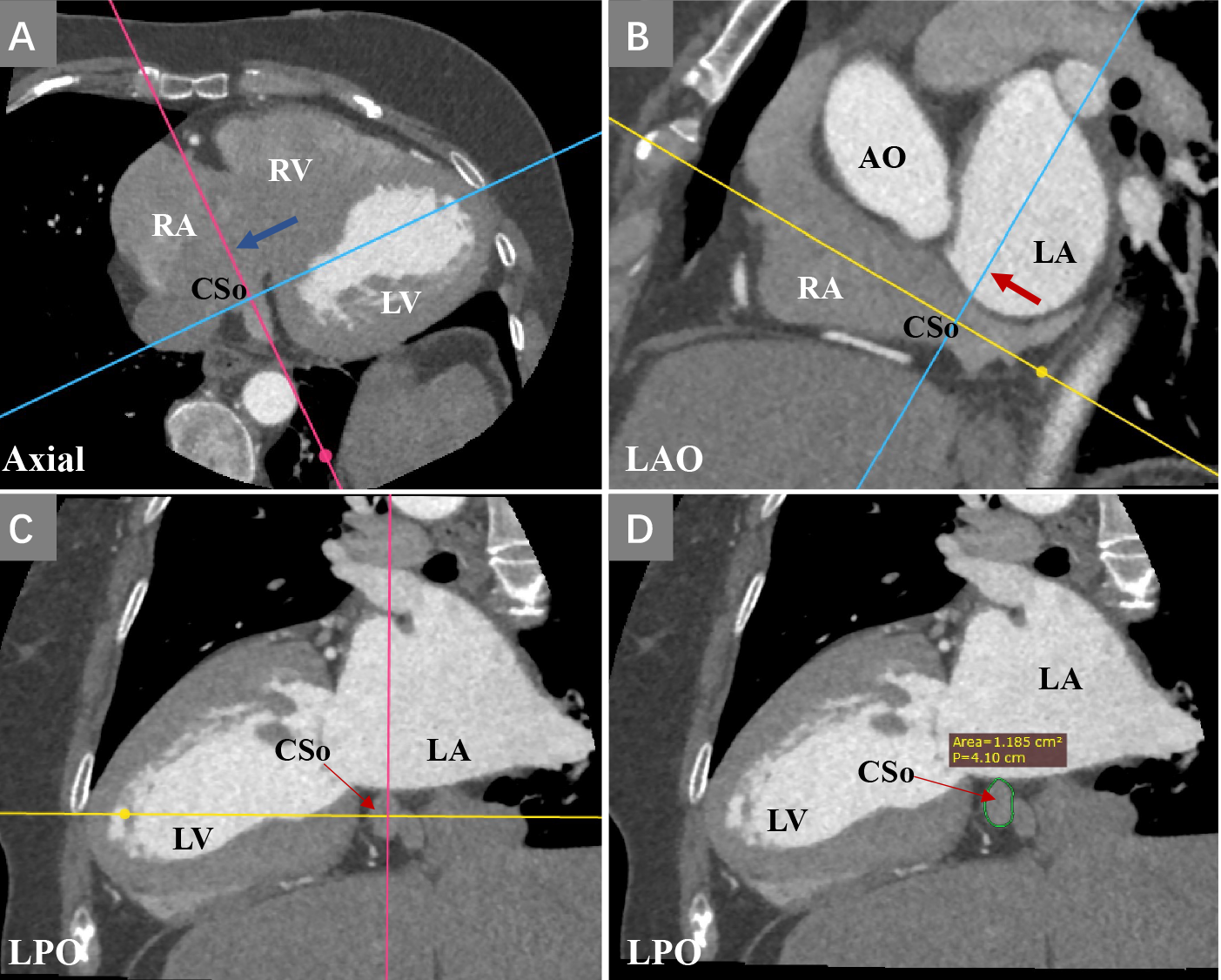

Supplement: Supplementary Figure 1 — Stepwise procedure of measurement of the CS ostium surface area. (A) Adjustment of the blue line parallel to the CS ostium in the axial plane; (B) Adjustment of the blue line parallel to the CS ostium in the LAO plane; (C) After multiplanar reconstruction (A,B), the CS ostium was at the intersection of the pink line and yellow line in the LPO plane; (D) The CS ostium was encircled by the green line, and the surface area in the green circle was automatically calculated by the software (1.185 cm2). AVNRT, atrioventricular nodal reentrant tachycardia; CSo, coronary sinus ostium; LA, left atrium; LAO, left anterior oblique; LPO, left posterior oblique; LV, left ventricle; PAH, pulmonary arterial hypertension; AO, aorta; RA, right atrium. The blue, pink, and yellow lines were perpendicular to each other. A solid blue arrow in panel (A) represents the projection direction of panel (B); a solid red arrow in panel (B) represents the projection direction of panels (C,D). [file Image_1.TIF]

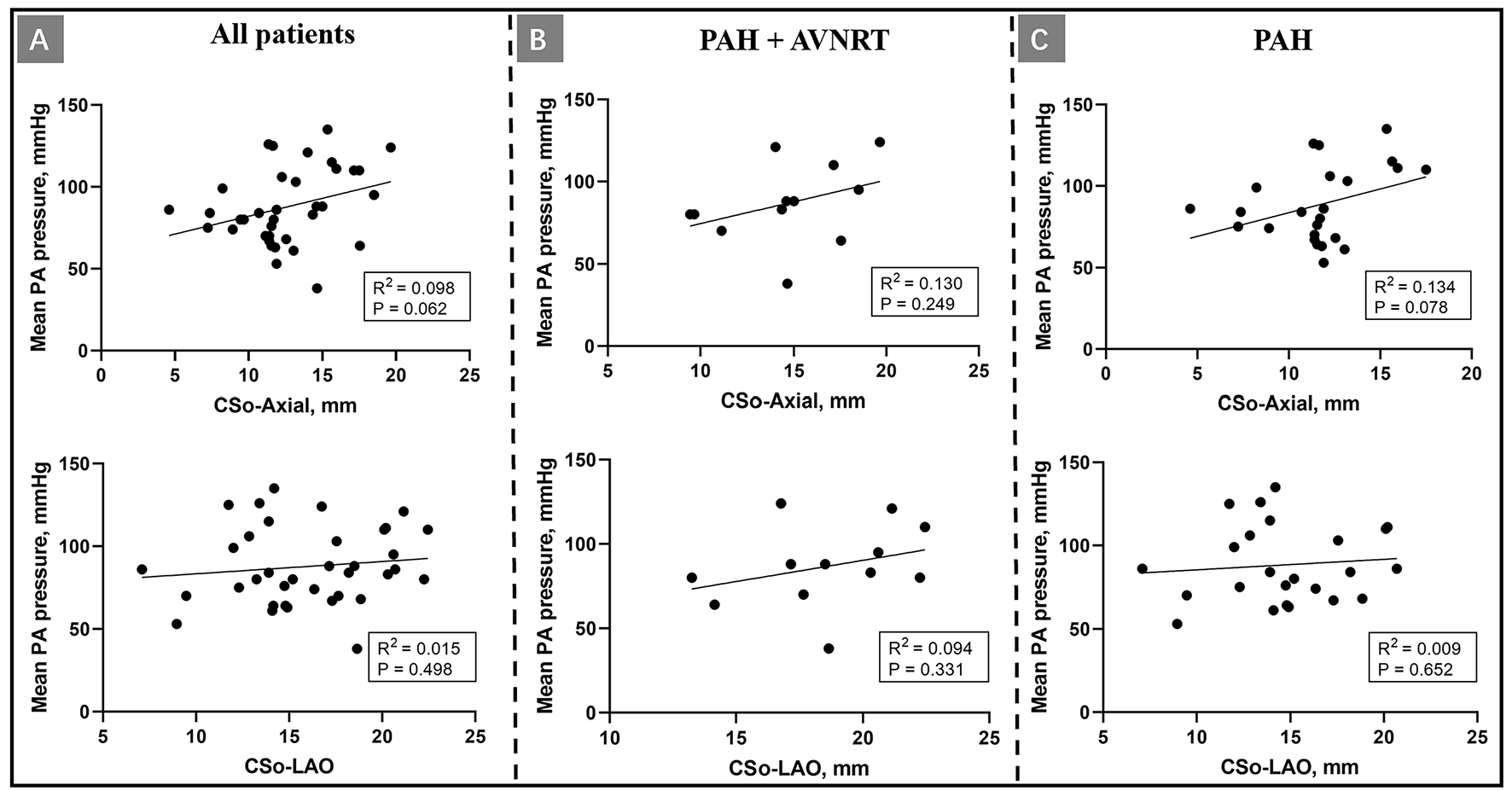

Supplement: Supplementary Figure 2 — (A) Correlation between the mean PA pressure and CS diameter in the LAO and axial planes in all patients (n = 36); (B) Correlation between the mean PA pressure and CS diameter in the LAO and axial plane in PAH patients with AVNRT (n = 12); (C) Correlation between the mean PA pressure and CS diameter in the LAO and axial planes (n = 24) in PAH patients. LAO, left anterior oblique; PA, pulmonary artery. [file Image_2.TIF]

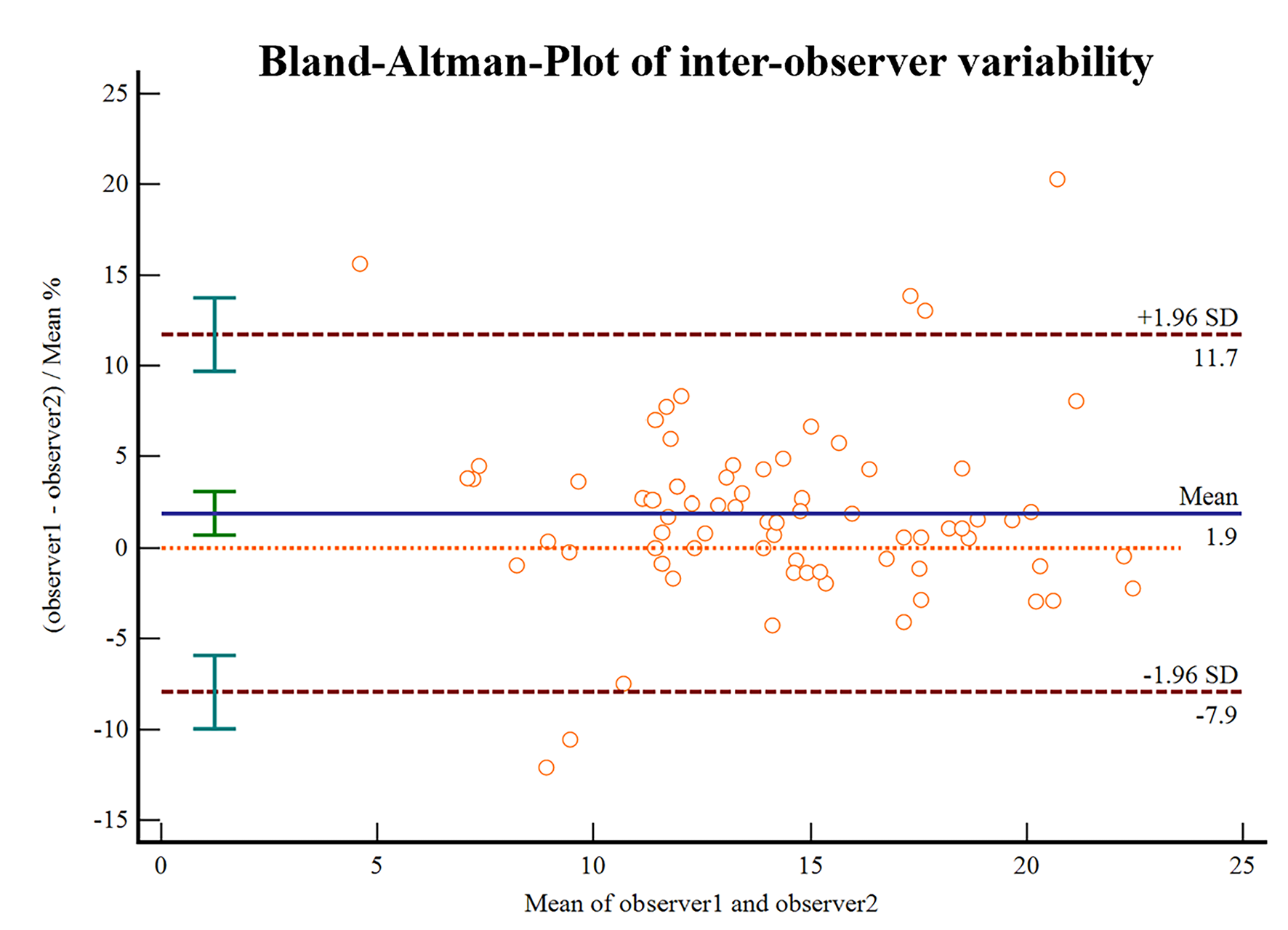

Supplement: Supplementary Figure 3 — Bland–Altman plots with 95% confidence intervals comparing measurements between observer 1 and observer 2. [file Image_3.TIF]

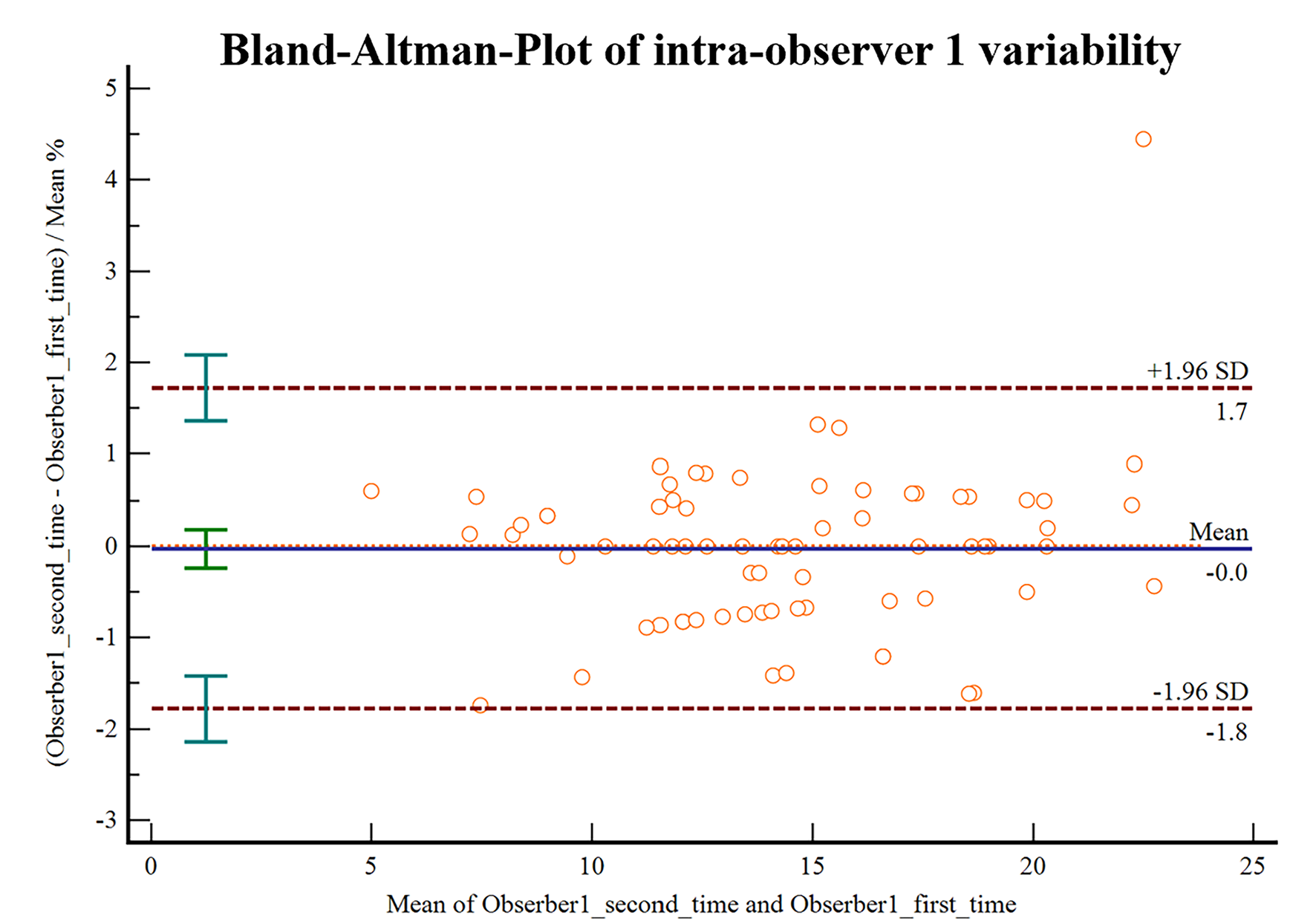

Supplement: Supplementary Figure 4 — Bland–Altman plots with 95% confidence intervals comparing measurements of observer 1 between the first time and the second time. [file Image_4.TIF]

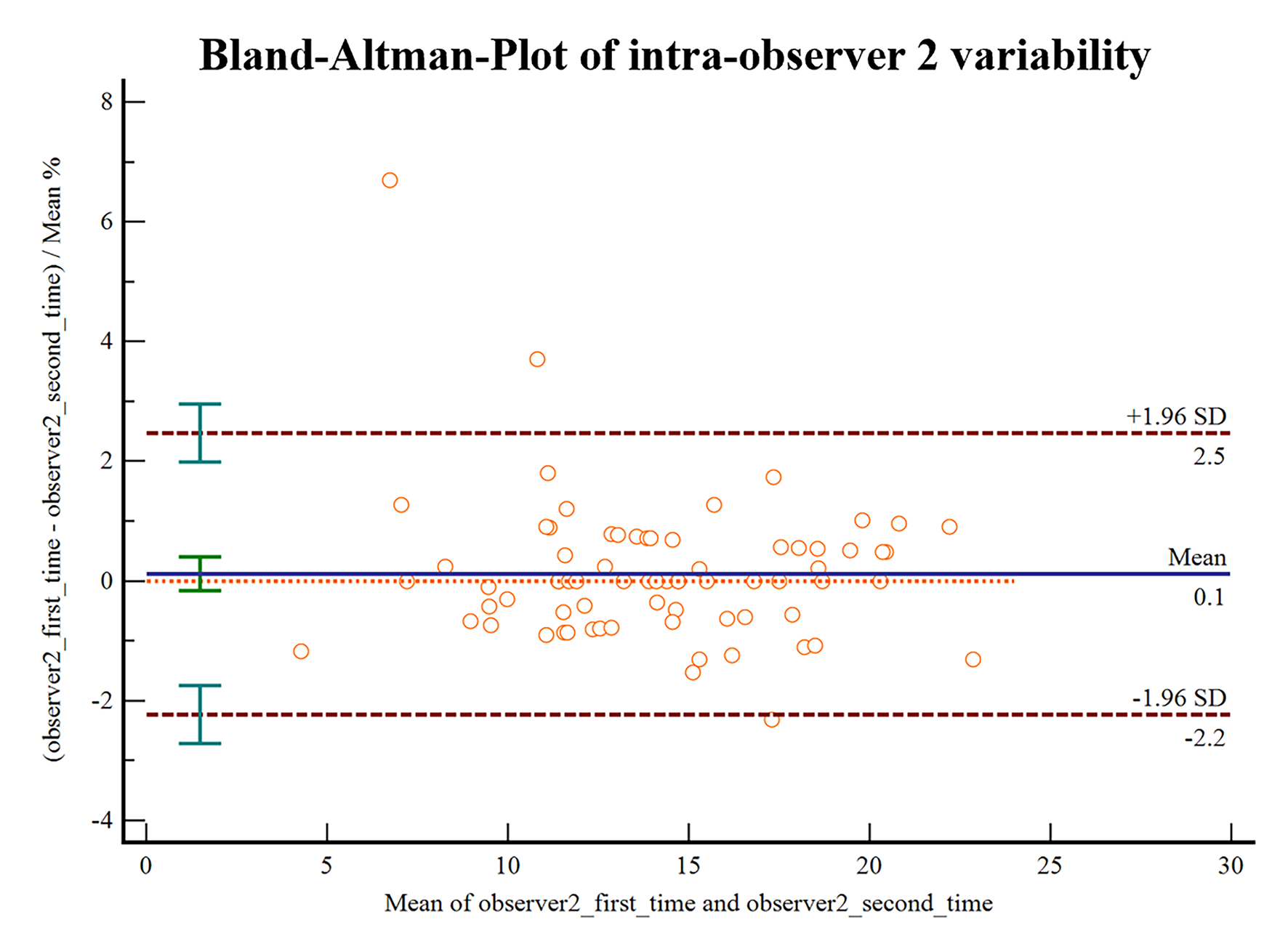

Supplement: Supplementary Figure 5 — Bland–Altman plots with 95% confidence intervals comparing measurements of observer 2 between the first time and the second time. [file Image_5.TIF]
