# Supplementary material for: Association Between the Coronary Sinus Ostial Size and Atrioventricular Nodal Reentrant Tachycardia in Patients With Pulmonary Arterial Hypertension
Source: Front Physiol. 2022 Jan 21;12:790077. doi: 10.3389/fphys.2021.790077 (PMC8814530; doi:10.3389/fphys.2021.790077)
Supplement: Supplementary file 6 [file Data_Sheet_1.PDF]

Response Table 1 Electrophysiological properties of PAH+AVNRT patients

Variables	PAH+AVNRT (n=12)
**AH (ms)**	92.0 ± 29.5
**TCL (ms)**	382.8 ± 75.4
**Atrial fractionation at the site of ablation**	5.2 ± 1.3

AH = atrial-His interval; AVNRT = atrioventricular nodal reentrant tachycardia; PAH = pulmonary arterial hypertension; TCL = tachycardia cycle length. AH was measured in sinus rhythm.

Response Table 2 Surface area of CS ostium between two groups

Variables	PAH+AVNRT (n=12)	PAH (n=24)	***P* value**
**CS ostium surface area (cm^2)**	2.08 ± 1.35	1.45 ± 0.73	0.039*

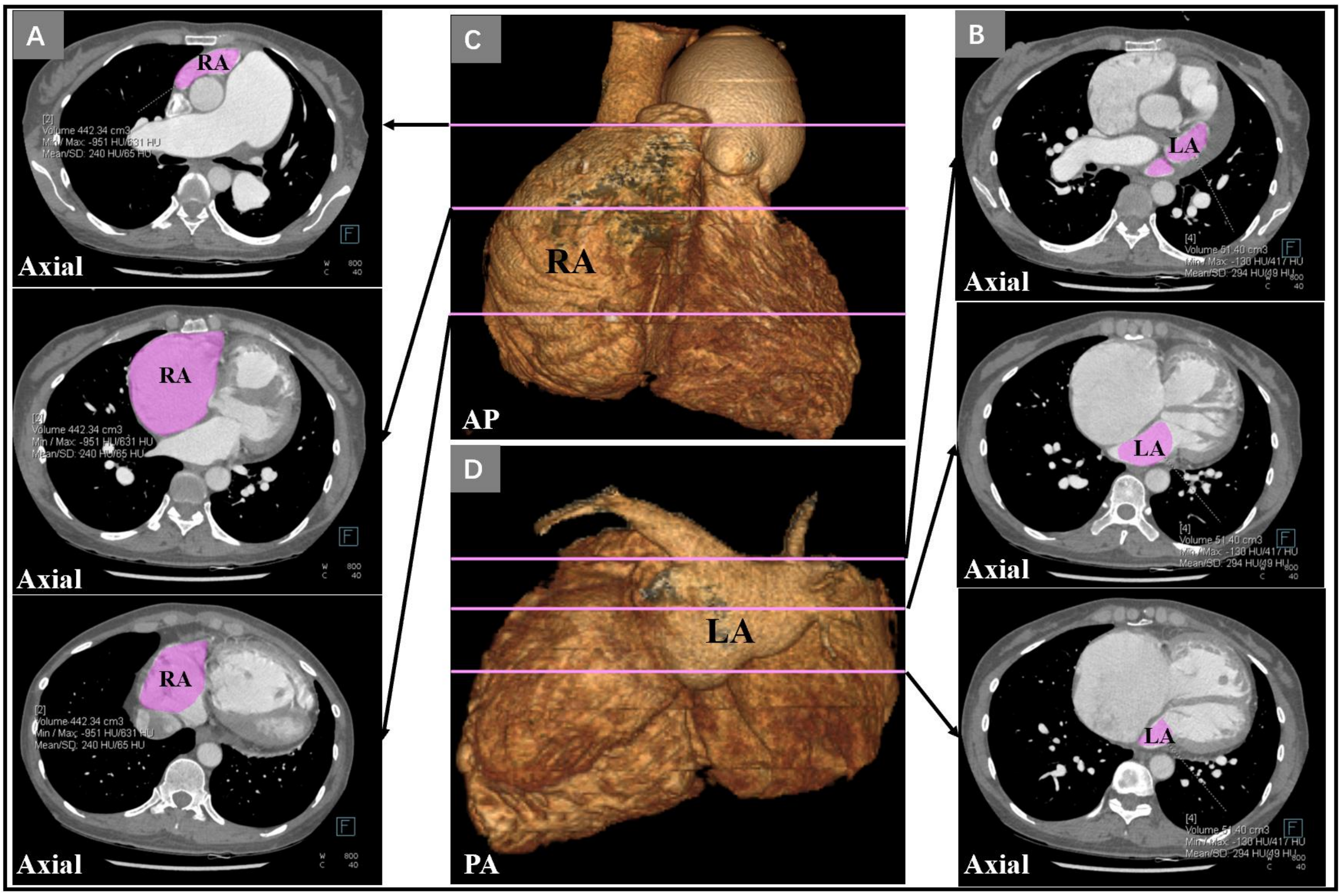

Response Figure 1 Measurement of atrium volume via CTA. (A) Representative slices of the high, middle, and low RA; (B) Representative slices of the high, middle, and low LA; (C) Anteroposterior position of the three-dimensional reconstruction of the heart; (D) Posteroanterior position of the three-dimensional reconstruction of the heart. The pink area represents the area of free-hand regions of interest. AP = anteroposterior position; LA = left atrium; PA = posteroanterior position; RA = right atrium.

We included Responses 2A and B in the manuscript as Figure 3A and B, and included Response Figures 3-5 as Supplementary Figures 3-5.

A

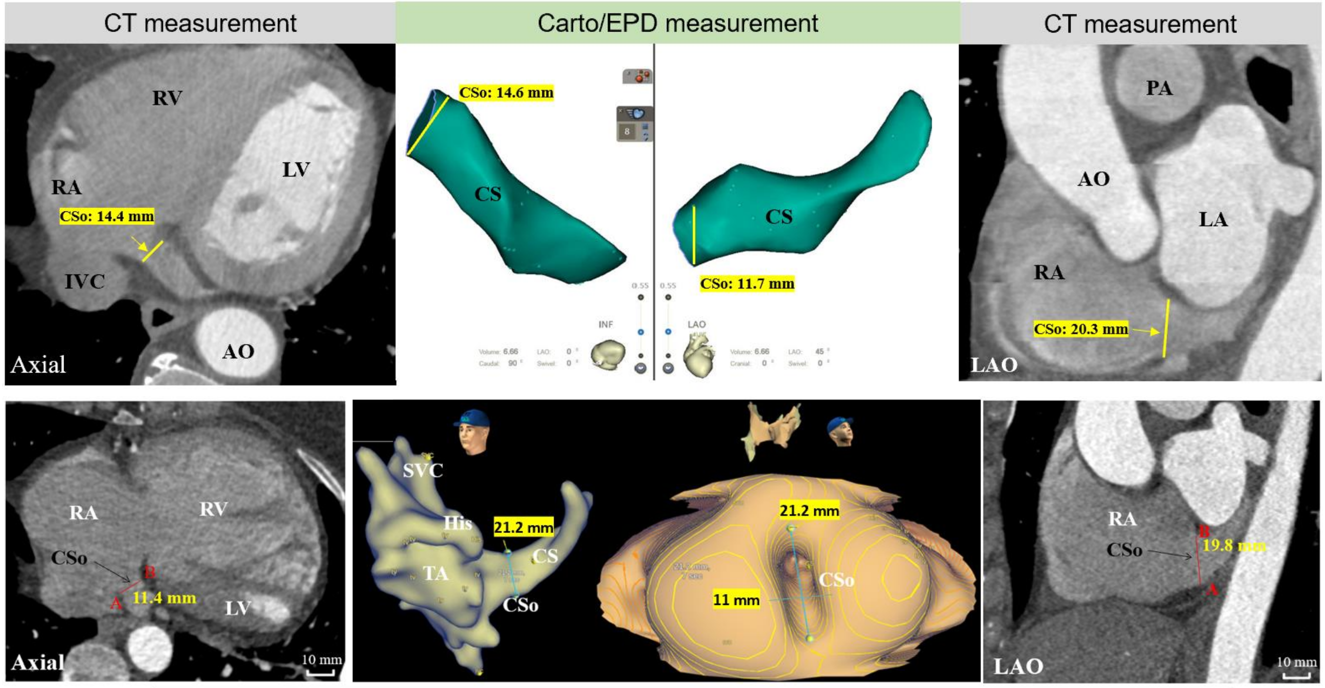

B

	Axial-CT /mm	LAO-CT /mm	Axial-CARTO /mm	LAO-CARTO /mm	Axial-EPD /mm	LAO-EPD /mm
Patient 1	14.4	20.3	14.6	**11.7**	-	-
Patient 2	11.2	17.7	**7.3**	**10.7**	-	-
Patient 3	11.4	19.8	-	-	11	21.2
Patient 4	16.1	20.5	-	-	**11.1**	20.6

Red represented the measuring error (compare with CT) great than 2 mm

Response Figure 6 (A) Measuring example. (B) Value of measurement among those 4 patients.
CS= coronary sinus; CSo = coronary sinus ostium; His = His bundle catheter; LAO = left anterior oblique; LV = left ventricle; PA = pulmonary artery; AO=aorta; RA = right atrium; RV = right ventricle; SVC = superior vena cava; IVC = inferior vena cava; TA = tricuspid annulus.

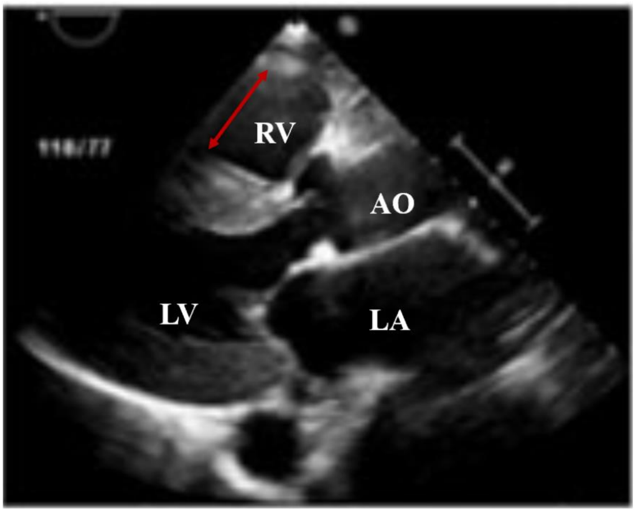

Response Figure 7 Measurement of RV dimensions in the parasternal long-axis view. Red arrow indicates the anteroposterior diameter of RV. Figure was cited from Circulation[1].
